# Percutaneous periarticular analgesic injection at one day after simultaneous bilateral total knee arthroplasty: an open-label randomized control trial

**DOI:** 10.1186/s13018-021-02507-1

**Published:** 2021-06-01

**Authors:** Takuya Iseki, Sachiyuki Tsukada, Motohiro Wakui, Kenji Kurosaka, Shinichi Yoshiya, Toshiya Tachibana

**Affiliations:** 1 Department of Orthopaedic Surgery, Nekoyama Miyao Hospital, 14-7 Konan, Chuo-ku, 950-1151 Niigata Japan; 2 Department of Orthopaedic Surgery, Hyogo College of Medicine, 1-1 Mukogawa-cho, Nishinomiya, Hyogo 663-8501 Japan; 3 Department of Orthopaedic Surgery, Houksuikai Kinen Hospital, 3-2-1 Higashihara, Mito, 310-0035 Ibaraki Japan; 4 Department of Orthopaedic Surgery, Nishinomiya Kaisei Hospital, 1-4 Ohama-cho, Nishinomiya, 662-0957 Hyogo Japan

**Keywords:** Simultaneous bilateral total knee arthroplasty, Postoperative pain, Additional injection, Randomized

## Abstract

**Background:**

The postoperative pain after total knee arthroplasty (TKA) remains a critical issue. The aim of this study was to assess the clinical effectiveness of percutaneous periarticular injection at 1 day following simultaneous bilateral TKA.

**Methods:**

A total of 88 knees in 44 patients who underwent simultaneous bilateral TKA were randomly assigned to receive a percutaneous periarticular injection at 1 day following surgery (n = 22 patients) or no injection (n = 22 patients). In the additional injection group, we injected a solution including methylprednisolone, ropivacaine, and epinephrine into the muscle belly of the vastus medialis at 1 day after surgery. In both groups, patients received an intraoperative periarticular multi-drug injection and postoperative intravenous and oral nonsteroidal anti-inflammatory drugs. The primary outcome measure was the postoperative pain at rest using a visual analog scale (VAS) and analyzed with Student’s *t* test.

**Results:**

Compared to the no additional injection group, the additional periarticular injection group had significantly lower VAS score at 8:00 PM postoperative day 1, 6:00 AM postoperative day 2, 12:00 PM postoperative day 2, 6:00 AM postoperative day 5, 12:00 PM postoperative day 5, and 8:00 PM postoperative day 5 (p < 0.05). The rate of complication did not differ between groups (p > 0.05).

**Conclusion:**

Additional percutaneous periarticular injection at 1 day following TKA adding to intraoperative periarticular injection provided better postoperative pain relief.

**Trial registration:**

Registered at the University Hospital Medical Information Network (registration number: UMIN000029759).

## Introduction

Pain management in the early period is one of the most important factors in TKA, because the intense postoperative pain in the early period after TKA affects patient satisfaction [1–3]. Intraoperative periarticular multi-drug injection has been reported to be effective in many studies as a multimodal pain management after TKA [4–7]. In simultaneous bilateral TKA, intraoperative periarticular multi-drug injection has also been reported to be effective [8–10]. However, one of the problems with intraoperative periarticular multi-drug injection was that the pain score gradually elevated at 24 h postoperatively, because the effect of intraoperative periarticular multi-drug injection gradually diminished [6, 11]. The rebounding pain after the early postoperative period is a critical issue for multimodal pain management after TKA [12]. According to a previous study, the early postoperative pain score at rest after unilateral TKA was lower in patients with the additional percutaneous periarticular multi-drug injection (additional injection) [13]. The concept of this additional injection was to add the already established intraoperative periarticular multi-drug injection once again at 1 day after TKA. However, there is no information of the efficacy of this additional injection for simultaneous bilateral TKA.

The purpose of this study was to examine the effects of additional injection at 1 day after simultaneous bilateral TKA. The hypothesis of this study is that the postoperative pain score would be lower in patients that received the additional injection at 1 day after simultaneous bilateral TKA.

## Materials and methods

### Study design

This study was a prospective, single-center, two-arm, parallel-group, open-label, and randomized controlled trial. The study protocol was approved by the institutional review board. All patients provided written informed consent to participate in the study. The study was registered as a randomized controlled trial with the University Hospital Medical Information Network (registration number: UMIN000029759. Registered 30 October 2017, https://upload.umin.ac.jp/cgi-open-bin/ctr/ctr_view.cgi?recptno=R000034000).

### Study population

Among the patients scheduled for simultaneous bilateral TKA from November 2017 to March 2018, those who have consented to participate in this study were eligible for inclusion. The exclusion criteria were patients scheduled for unilateral TKA, staged bilateral TKA, revision TKA, TKA combined with implant removal, and patients with allergies to drugs used in the study. Patients who had poorly controlled diabetes mellitus defined as hemoglobin A1c with levels over 7.0% were also excluded because they did not receive steroids in the intraoperative periarticular multi-drug injection.

### Randomization

We randomly assigned the eligible patients to receive additional percutaneous periarticular injection at 1 day after simultaneous bilateral TKA (additional injection group) or no injection at 1 day after TKA (control group). The randomization sequence was created by permuted block randomization with a block size of 4 and a 1:1 allocation generated using computer software R, (The R Foundation for Statistical Computing). The eligible patients received a generated randomized number accordingly. Patients with even numbers were allocated to the additional injection group and those with odd numbers were allocated to the control group.

### Interventions

**Fig. 1:**
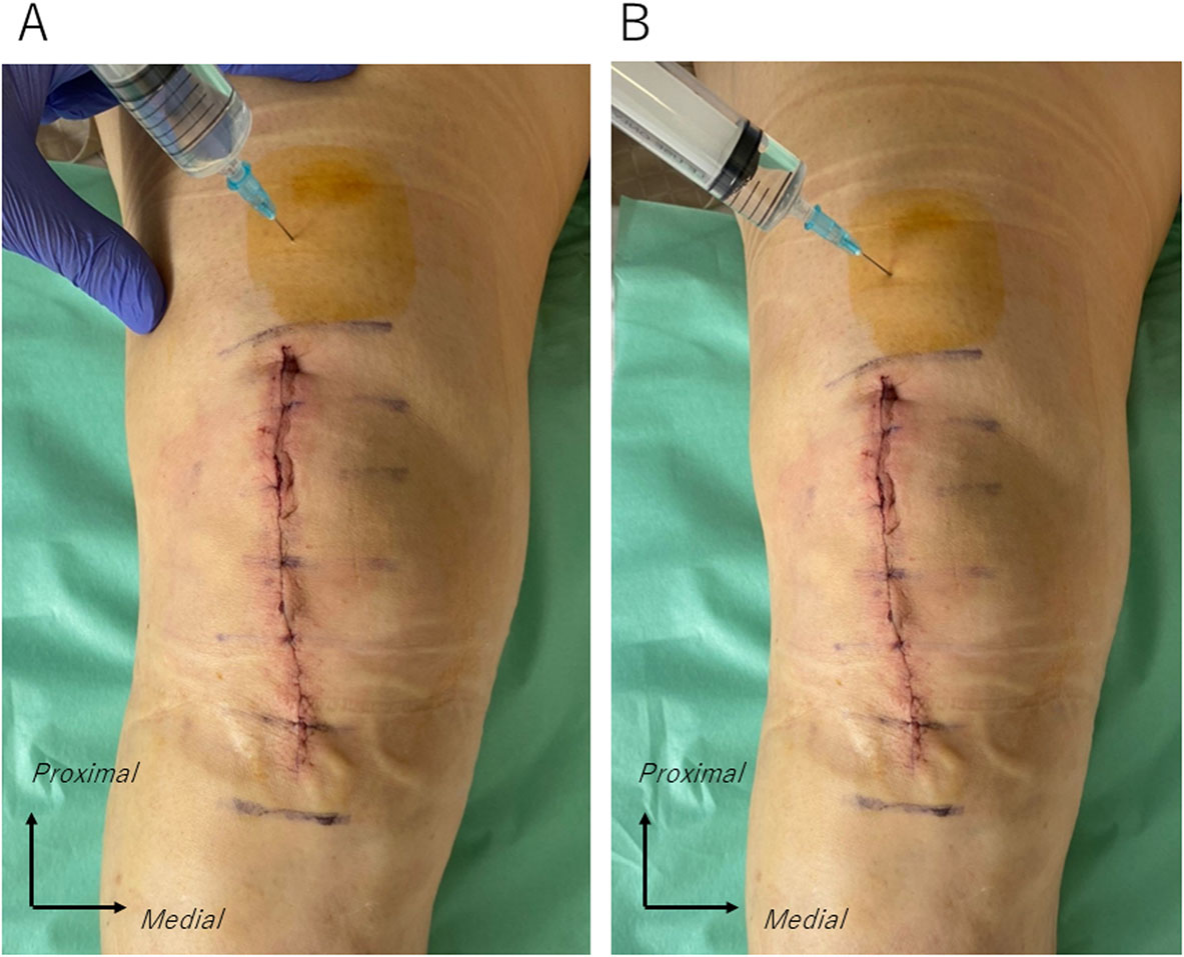
Percutaneous periarticular analgesic injection at 1 day after surgery. (For right knee.) **a** Inject 6.1 ml of drug solution into muscle belly of the vastus medialis just medial to the quadriceps tendon. **b** Direct the tip of the needle inward and inject 5 ml of drug solution into the muscle belly of the vastus medialis at a more medial site than the first infiltration.

The additional injection was routinely administered at 8:30 AM the day after surgery regardless of the time TKA was performed. In the additional injection group, a total of 22.2 mL of percutaneous periarticular multi-drug injection, including methylprednisolone 80 mg [2 mL], ropivacaine 150 mg [20 mL], and epinephrine 0.2 mg [0.2 mL] was prepared, and divided into 11.1 mL and each injected into the right knee and the left knee. The additional injection technique was the same as we previously reported [13]. We percutaneously injected the analgesic solution at approximately 3 cm above the superior border of the antero-medial skin incision, using a 23-gauge needle (Fig. 1). First, we infiltrated 6.1 mL of solution into the muscle belly of the vastus medialis just medial to the quadriceps tendon (Fig. 1A). Second, we moved the needle tip and infiltrated the remaining 5 mL of solution into the muscle belly of vastus medialis at a more medial site than the first infiltration (Fig. 1B). After the injection of one knee, the other knee was injected in the same manner.

### Perioperative medication

All patients received intraoperative periarticular injections. As an intraoperative periarticular injection, methylprednisolone 80 mg (Sol Mercort; Fuji, Toyama, Japan) [2 mL], 7.5 mg/mL ropivacaine (Anapeine; AstraZeneca, Osaka, Japan) [40 mL], 10 mg/mL morphine hydrochloride hydrate (Takeda, Osaka, Japan) [1.0 mL], 1.0 mg/mL epinephrine (Bosmin; Daiichi-Sankyo, Tokyo, Japan) [0.6 mL], 50 mg of ketoprofen (Capisten; Kissei, Matsumoto, Japan) [2.5 mL], and normal saline [13.9 mL] were routinely injected in both groups. All patients received a non-steroidal anti-inflammatory drug (50 mg of flurbiprofen axetil, Ropion; Kaken, Tokyo, Japan) intravenously 1 h after returning to the ward, and non-steroidal anti-inflammatory drug (60 mg of loxoprofen, Surinofen; Aska, Tokyo, Japan) was administered orally three times a day from 1 day after surgery. As rescue analgesic medication, a 25 mg of diclofenac sodium suppository (Adefuroniczupo; Teva, Nagoya, Japan) was used. No narcotic pain medications were used in postoperative medication.

### Surgery and rehabilitation

All surgical procedures were performed by one of three surgeons (IT, ST, and MW) using spinal anesthesia with 2.4 to 3.2 mL of 0.5% bupivacaine (Marcaine; AstraZeneca). We did not use the pneumatic tourniquet or drain for any patients. Anteromedial straight skin incision of 4 cm proximal to the superior patella to 1 cm distal to the tibial tuberosity was made, and subvastus approach were used in all surgeries. All patients received a cemented, posterior stabilized prosthesis (Scorpio NRG; Stryker Orthopedics, Mahwah, NJ, USA).

The postoperative rehabilitation regimens were the same for both groups and were started from 1 day after surgery in the afternoon.

### Outcome measurements

#### Primary outcome

We evaluated the pain scale at rest for each knee using the VAS score as a primary outcome of this study. The VAS score ranged from 0 mm (indicating no pain) to 100 mm (indicating extreme pain). The VAS score was measured for each knee at 12:00 PM and 8:00 PM at 1 day after surgery. From 2 to 5 days after surgery, the VAS score was measured at 6:00 AM, 12:00 PM, and 8:00 PM. The measurements of VAS score were completed by the patient and confirmed by the nurse at all time points. In addition, we assessed whether the VAS score at rest in both groups achieved the patient acceptable symptom state (PASS) and the differences in the VAS score at rest between the two groups reached minimum clinically important difference (MCID). The threshold of PASS was defined as 33 mm according to the study conducted by Myles et al. [14]. The threshold of MCID was determined as 20 mm [8, 11].

#### Secondary outcome

The secondary outcome of this study included the VAS score during activity, range of motion of the knee, and complications. The VAS score during activity was defined as the strongest pain experienced during physical therapy. Consumption of rescue analgesia was also recorded during the study period. The data was collected from postoperative days 1 to 5.

#### Sample size

As for sample size, 19 patients per group were calculated to illustrate the MCID of 20 mm in the VAS score at rest [8, 11], with a two-sided 5% significance level and 80% power. For power analysis, we used a standard deviation of 22 mm in the VAS score based on the results of our pilot series.

### Statistical analysis

All statistical analyses were performed using R software. We replaced missing values of the VAS score at rest either by linear interpolation in cases where the missing values fell between two valid values or by median values from other patients at the same time point in the same treatment group. Comparisons between the study groups were performed using Student’s *t* test for continuous variables and the chi-square test for categorical variables, respectively. All tests were two-sided, and *P* < 0.05 was considered statistically significant.

## Results

### Participants

**Fig. 2:**
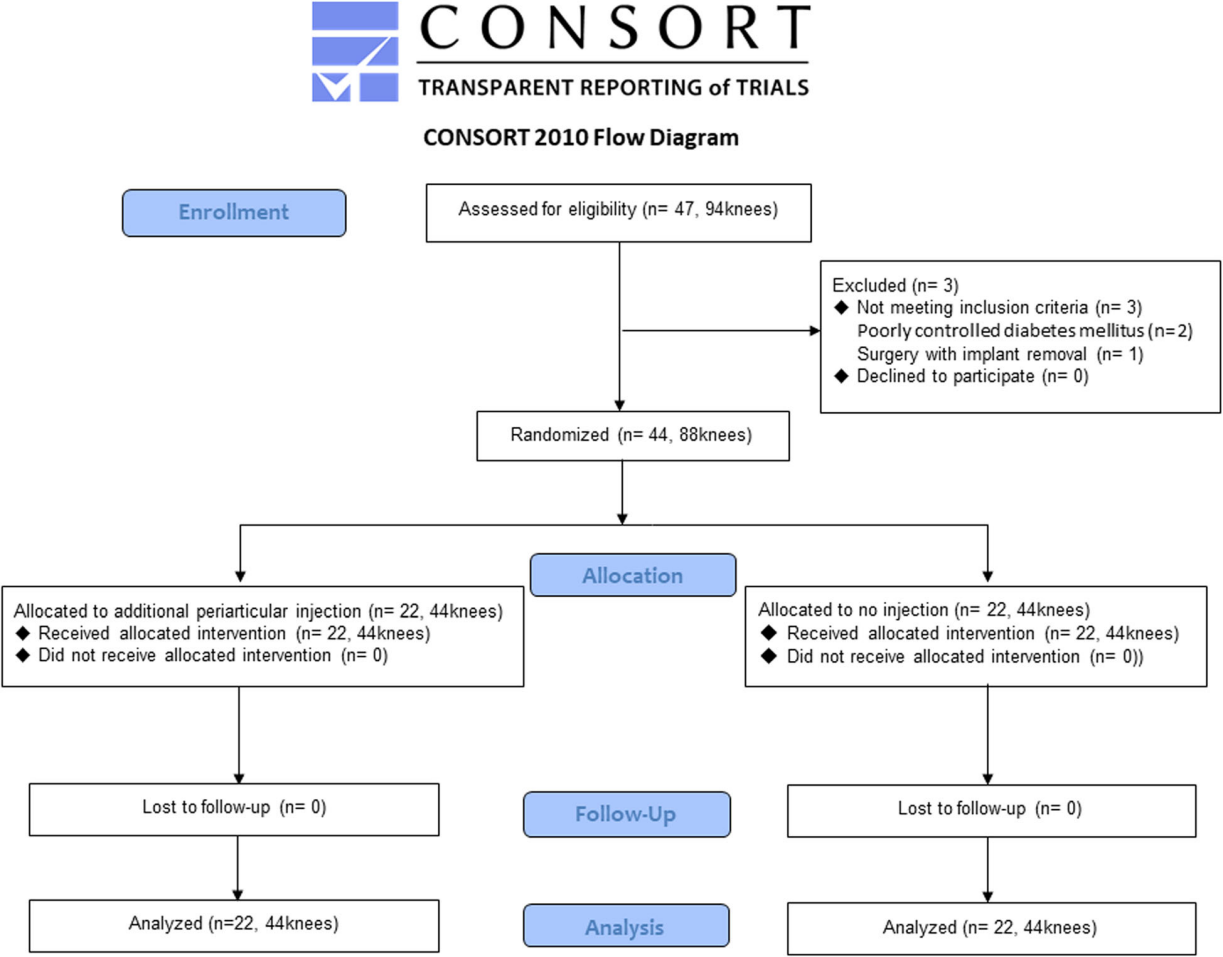
Diagram showing the flow of patients through each stage of the trial

The flowchart presented in Fig. 2 outlines the trial. A total of 94 knees in 47 patients underwent simultaneous bilateral TKA during the study period and 44 patients were randomly assigned to receive an additional injection (n = 22) or no injection (n = 22). One patient in the additional injection group had avascular necrosis in the medial femoral condyle on both sides. Table 1 summarizes the demographic characteristics of the patients and there were no significant differences between the two groups.

**Table 1:** Patient demographics and baseline clinical characteristics

	Additional injection (22 patients/44 knees)	Control (22 patients/44 knees)	***P*** value
Age (years)	77 ± 7	76 ± 7	0.78
Sex (female/male)	19/3	15/7	0.28
Height (cm)	151 ± 5	151 ± 9	0.94
Weight (kg)	59.2 ± 9.1	61.1 ± 9.6	0.50
BMI (kg/m^2)^	25.8 ± 3.2	26.7 ± 3.5	0.34
Diagnosis (OA/AN)	21/1	22/0	1.00
History of diabetes mellitus (yes/no)	3/19	4/18	1.00
Preoperative VAS at rest (mm)	38 ± 24	32 ± 26	0.26
Preoperative VAS during activity (mm)	46 ± 23	47 ± 24	0.86
Preoperative flexion angle (d)	121 ± 18	119 ± 15	0.74
Preoperative extension angle (d)	− 7 ± 6	− 9 ± 7	0.29
Surgeon (TI/ST/MW)	11/8/3	12/7/3	0.95
Duration of surgery (month)	159 ± 24	157 ± 24	0.80

*Abbreviations*: *BMI* body mass index, *OA* osteoarthritis, *AN* avascular necrosis, *VAS* visual analog scale

*Note*: Results are expressed as means ± standard deviation, unless stated otherwise

### Primary outcome

**Fig. 3:**
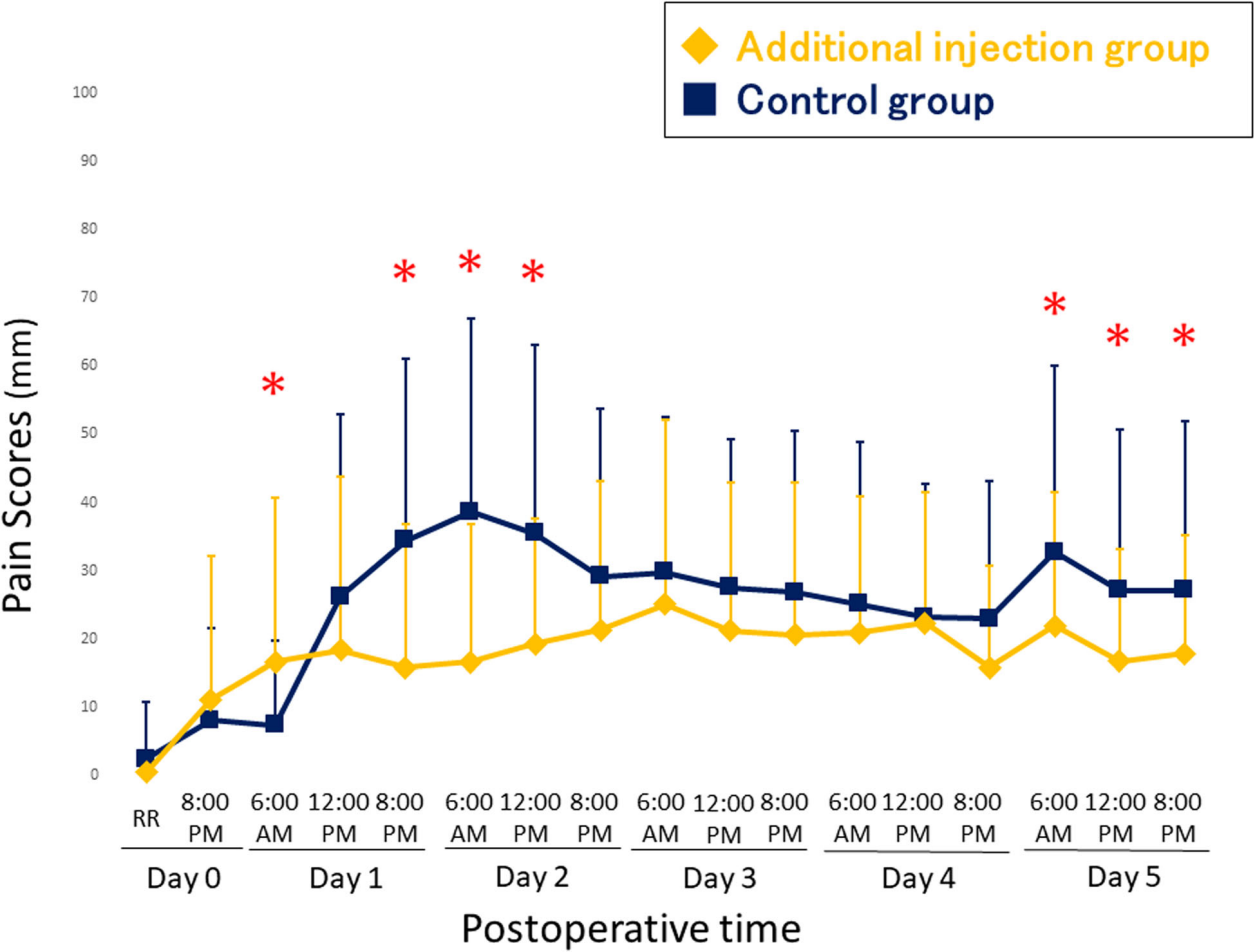
Visual analogue scale score of postoperative pain at rest. * p < 0.05

The pain VAS score at rest is shown in Table 2 and Fig. 3. The VAS score at 6:00 AM postoperative day 1 before the additional injection was lower in the control group. However, the additional injection group had significantly lower VAS score at 8:00 PM postoperative day 1 than the control group. Additionally, the pain VAS score at 6:00 AM postoperative day 2, and 12:00 PM postoperative day 2 were also lower in the additional injection group. The pain VAS score at 6:00 AM postoperative day 5, at 12:00 PM postoperative day 5, and 8:00 PM postoperative day 5 were also lower in the additional injection group.

**Table 2:** Visual analog scale score of postoperative pain at rest

Duration after surgery	Additional injection (22 patients/44 knees)	Control (22 patients/44 knees)	***P*** value
Day 0, recovery room	1 ± 2	2 ± 8	0.12
Day 0, 08: 00 PM	11 ± 21	8 ± 13	0.43
Day 1, 06:00 AM	17 ± 24	7 ± 12	0.026^a^
Day 1, 12:00 PM	18 ± 25	26 ± 27	0.16
Day 1, 08:00 PM	16 ± 21	34 ± 27	< 0.001^a^
Day 2, 06:00 AM	17 ± 20	39 ± 28	< 0.001^a^
Day 2, 12:00 PM	19 ± 18	35 ± 28	0.0019^a^
Day 2, 08:00 PM	21 ± 22	29 ± 24	0.13
Day 3, 06:00 AM	25 ± 27	30 ± 23	0.40
Day 3, 12:00 PM	21 ± 22	28 ± 22	0.18
Day 3, 08:00 PM	21 ± 22	27 ± 24	0.22
Day 4, 06:00 AM	21 ± 20	25 ± 24	0.39
Day 4, 12:00 PM	22 ± 19	23 ± 20	0.84
Day 4, 08:00 PM	16 ± 15	23 ± 20	0.068
Day 5, 06:00 AM	22 ± 20	33 ± 27	0.039^a^
Day 5, 12:00 PM	17 ± 16	27 ± 24	0.020^a^
Day 5, 08:00 PM	18 ± 17	27 ± 25	0.047^a^

*Note*: The additional injection was routinely performed at 08:30 AM the day after surgery regardless of the time that total knee arthroplasty was performed

Results are expressed as mean ± standard deviation, unless stated otherwise

^a^Significant at *p* < 0.05

In the control group, the mean pain VAS score at rest exceeded the threshold value of the PASS of 33mm in the early postoperative period, at 8:00 PM postoperative day 1, at 6:00 AM postoperative day 2, and at 12:00 PM postoperative day 2. In the additional injection group, on the other hand, the mean pain VAS score at rest was below the threshold value of the PASS of 33 mm at all assessment time points after TKA. The difference between the two groups reached the MCID of 20mm at 6:00 AM, postoperative day 2. The mean VAS scores at postoperative day 2, 6:00 AM were 17 mm in the additional injection group and 39 mm in the control group, respectively.

### Secondary outcome

**Fig. 4:**
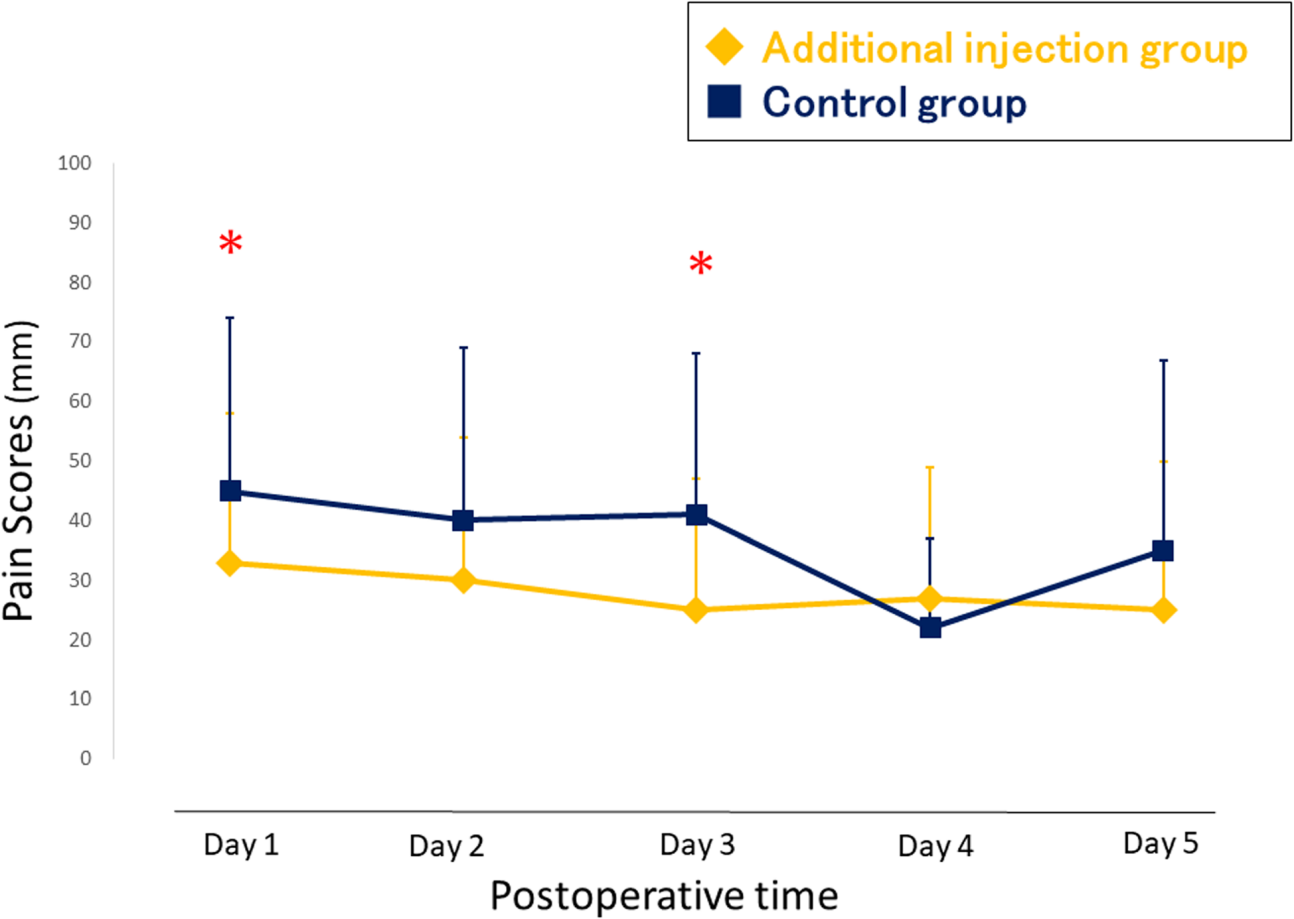
Visual analogue scale score of postoperative pain during activity. * p < 0.05

**Fig. 5:**
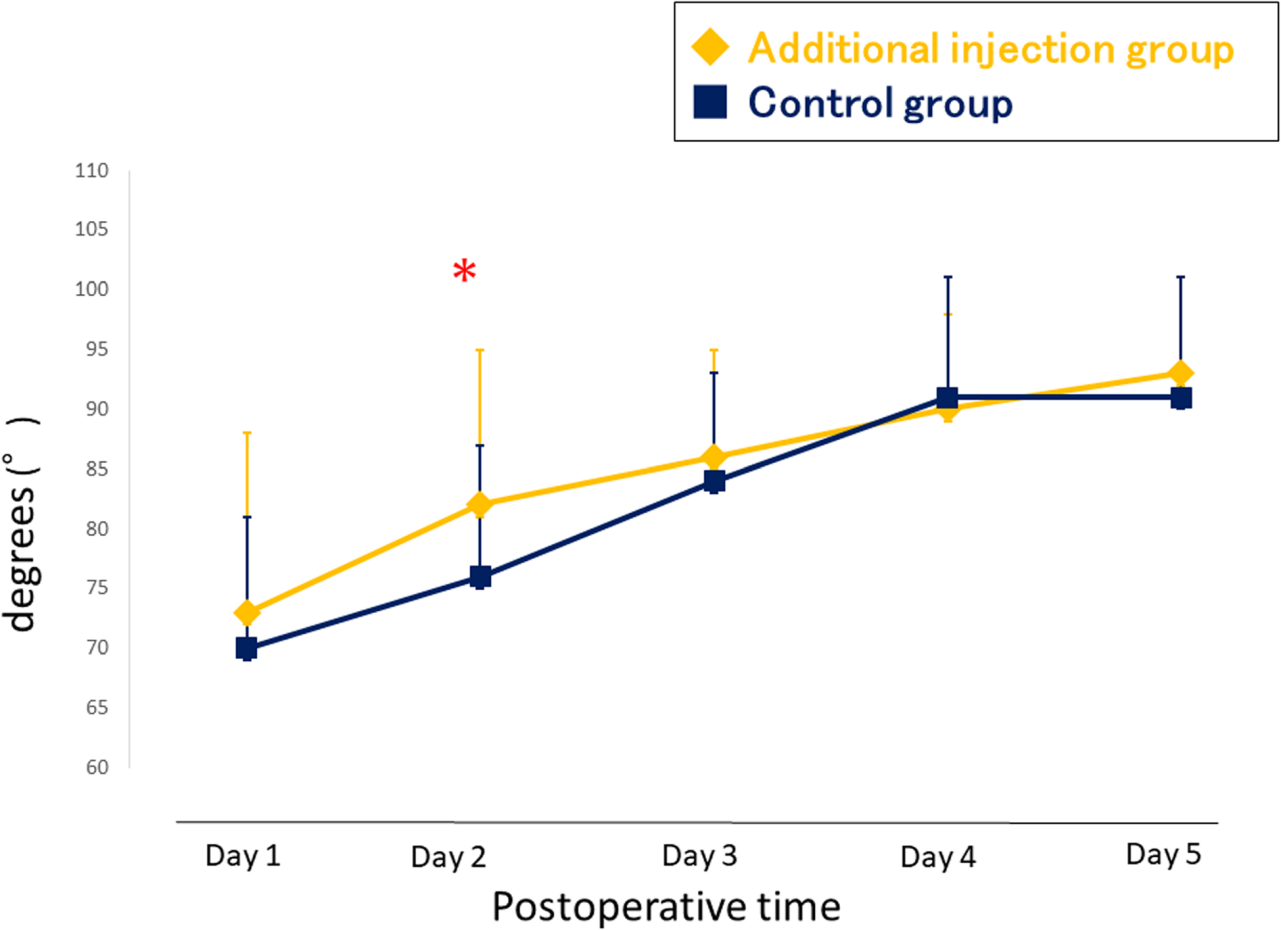
Postoperative flexion angle of the knee. * p < 0.05

**Fig. 6:**
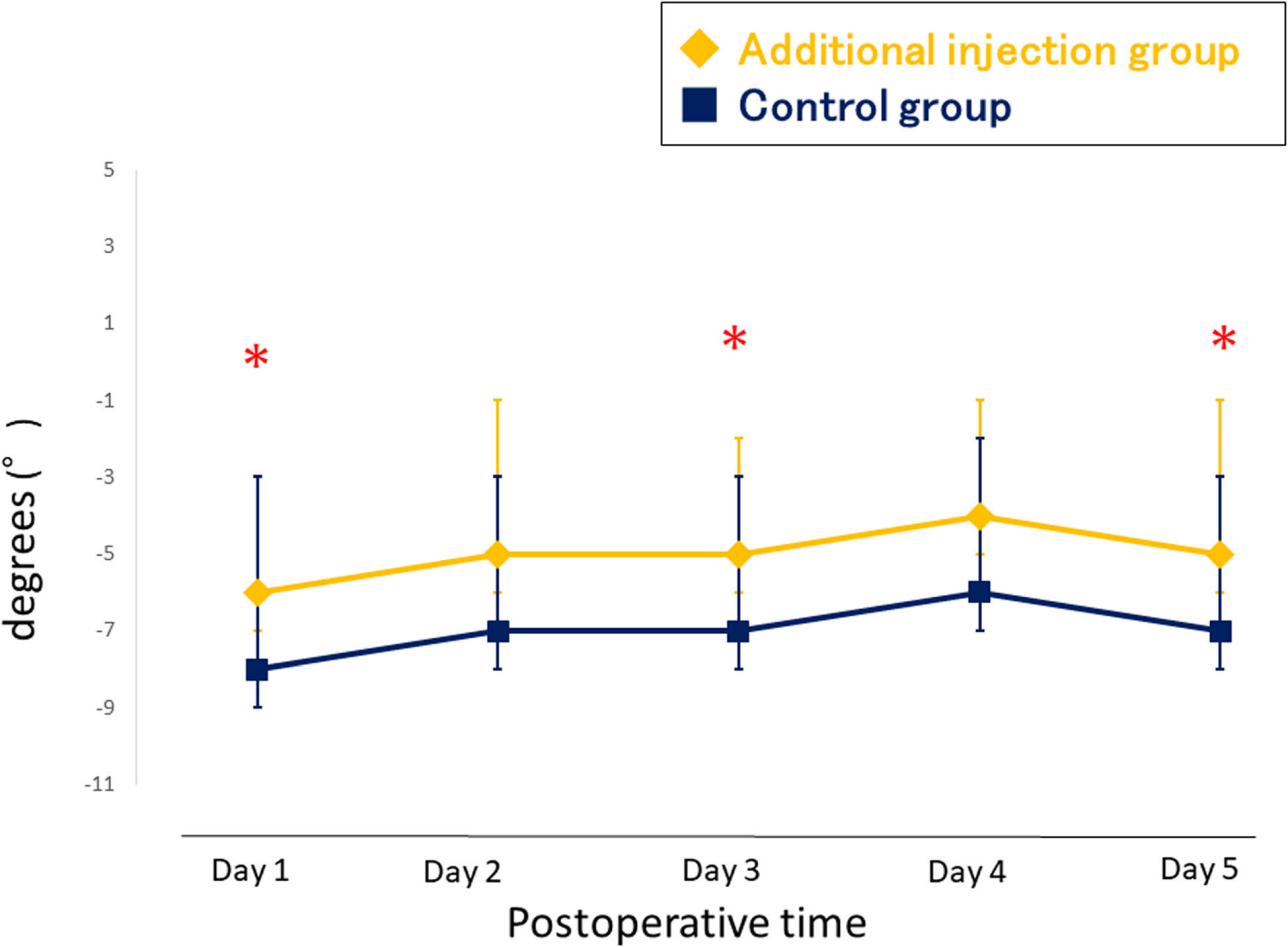
Postoperative extension angle of the knee. * p < 0.05

The pain VAS scores during activity are shown in Fig. 4. The additional injection group had significantly lower VAS scores at 1 day and 3 days after TKA. The consumption of diclofenac sodium suppository as rescue analgesia was similar between the two groups (Table 3). The flexion angle of the knee was better in the additional injection group at postoperative day 2 (Fig. 5). The extension angle of the knee was better in the additional injection group at postoperative day 1, 3, and 5 (Fig. 6). There were no wound complications nor surgical site infections in any study patients at the time of 2-year follow-up after surgery.

**Table 3:** Mean number of suppositories used as rescue analgesia

Duration after surgery	Additional injection (22 patients/44 knees)	Control (22 patients/44 knees)	***P*** value
On the night of surgery	0	0.09 ± 0.30	0.16
Day 1	0.23 ± 0.53	0.18 ± 0.39	0.75
Day 2	0.18 ± 0.39	0.32 ± 0.65	0.40
Day 3	0.23 ± 0.43	0.05 ± 0.21	0.082
Day 4	0.05 ± 0.21	0.09 ± 0.29	0.56
Day 5	0.14 ± 0.35	0.09 ± 0.29	0.64

Results are expressed as mean ± standard deviation, unless stated otherwise

## Discussion

The most important finding of this study was that additional injection including methylprednisolone, ropivacaine, and epinephrine at 1 day after simultaneous bilateral TKA reduced postoperative pain score. In this study, the pain VAS scores at rest were significantly lower at 8:00 PM postoperative day 1, at 6:00 AM postoperative day 2, and at 12:00 PM postoperative day 2 in the patients who received additional injection at 1 day after simultaneous bilateral TKA. Additionally, the pain VAS scores during activity on postoperative day 1 and day 3 were also lower in the additional injection group.

Recently, some studies have reported that approximately 20% of patients are not satisfied after TKA [15–17] and one of the causes is intense pain immediately after TKA. The early postoperative pain management is an important factor for patient satisfaction [1–3]. There are various reports on pain control in the early period after TKA. The femoral nerve block offers effective postoperative pain control [18]. However, continuous femoral nerve block causes weakness in the quadriceps muscle strength and could induce the risk of falling among other problems [19, 20]. Ikeuchi et al. reported the efficacy of local infusion analgesia using an intra-articular catheter after TKA [21] and Meier et al. reported the efficacy of local infiltration analgesia combined with a continuous additional postoperative intra-articular perfusion [22]. However, these methods have the disadvantage of requiring catheter placement after surgery. Ali et al. reported that the use of the continuous intraarticular infusion technique increased the rate of surgical site infection [23]. In the USA, excessive postoperative opioid prescriptions have been a problem after surgery. Patients who have undergone TKA especially tend to receive the highest amount of opioid medication [24–26].

The advantages of the additional percutaneous periarticular multi-drug injection at 1 day after TKA include not requiring procedures by anesthesiologists or a replacement of a catheter. There were no wound complications nor surgical site infections in the patients who received an additional injection, which suggests that this technique might be safe and uncomplicated for the management of pain after surgery. Additionally, this technique did not require the use of opioids in our regimen. We believe that our non-opioid pain management may be an alternative regimen to the pain relief after TKA. We have investigated the efficacy of percutaneous periarticular multi-drug injection at 1 day after unilateral TKA and concluded that it was effective for early postoperative pain management [13]. In this study, the difference of the mean VAS score at rest between two groups reached the MCID of 20 mm only at 6:00 AM postoperative day 2. However, at 8:00 PM postoperative day 1 and at 12:00 PM postoperative day 2, the difference in the mean VAS score at rest was close to MCID of 20 mm. We believe that it is clinically important to be able to reduce pain during this peak period of rebounding pain.

There were several limitations in this study. First, the treatment team members and patients were not blinded in this study. In addition, the control group not using placebo was also a limitation. Second, the sample size was too small to conclude the ratio of complications, including wound complications and surgical site infections, owing to their low frequency. Third, the additional injection was routinely performed at 08:30 AM postoperative day 1, regardless of the time TKA was performed. Thus, the periods of time between the completion of TKA and the injection were not standardized among study patients. Fourth, prior to commencing RCT, we determined to use block randomization in which treatment allocation was made without regard to prior allocation. Thus, we avoided adjusting confounders using stratified randomization. Unfortunately, the mean VAS score at 6:00 AM postoperatively day 1 was significantly different between two groups. However, we believe that the block randomization is still reasonable for this RCT because it can be greatly attributable to the issue of unmeasurable confounders. Fifth, postoperative quadriceps muscle strength was not measured. Finally, we did not assess long-term patient satisfaction.

## Conclusion

The additional percutaneous periarticular multi-drug injection at 1 day after simultaneous bilateral TKA may reduce early postoperative pain more effectively than no additional injection. This technique may be a new method for postoperative pain control after TKA.

## Data Availability

The datasets used and/or analyzed during the current study are not publicly available. Data are however available from the corresponding author on reasonable request.

## Abbreviations

MCID: Minimum clinically important difference
PASS: Patient acceptable symptom state
TKA: Total knee arthroplasty
VAS: Visual analog scale
